# Supplementary material for: Validation of the UNESP-Botucatu pig composite acute pain scale (UPAPS)
Source: PLoS One. 2020 Jun 1;15(6):e0233552. doi: 10.1371/journal.pone.0233552 (PMC7263847; doi:10.1371/journal.pone.0233552)
Supplement: S1 Table — (DOCX) [file pone.0233552.s001.docx]

**S1 Table – Medians (range) of the score of each item of the UPAPS assessed at the 2^nd^ phase of video analysis by the gold standard observer.**

| **Item** | **M1** | **M2** | **M3** | **M4** |
| --- | --- | --- | --- | --- |
| Posture | 0^c^ (0 – 0) | 2^a^ (0 – 3) | 0^c^ (0 – 1) | 1^b^ (0 – 2) |
| Interaction and interest in the surroundings | 0^b^ (0 – 0) | 1^a^ (0 – 3) | 0^b^ (0 – 1) | 0^a^ (0 – 2) |
| Activity | 0^b^ (0 – 0) | 1^a^ (0 – 3) | 0^b^ (0 – 1) | 0^a^ (0 – 2) |
| Appetite | 0^b^ (0 – 0) | 0^a^ (0 – 3) | 0^b^ (0 – 0) | 0^ab^ (0 – 2) |
| Attention to the affected area | 0^c^ (0 – 0) | 2^a^ (0 – 3) | 0^c^ (0 – 1) | 1^b^(0 – 3) |
| *A – elevates pelvic limb* | 0^b^ (0 – 0) | 0^ab^ (0 – 1) | 0^b^ (0 – 0) | 0^a^ (0 – 1) |
| *B – scratches/rubs the painful area* | 0 (0 – 0) | 0 (0 – 1) | 0 (0 – 0) | 0 (0 – 1) |
| *C – moves and/or runs away and/or jumps after injury of the lesion* | 0^b^ (0 – 0) | 0^a^ (0 – 1) | 0^b^ (0 – 1) | 0^ab^ (0 – 1) |
| *D – sits with difficulty* | 0^b^ (0 – 1) | 0^a^ (0 – 1) | 0^b^ (0 – 1) | 0^ab^ (0 – 1) |
| Miscellaneous | 0^b^ (0 – 1) | 2^a^ (0 – 3) | 0^b^ (0 – 2) | 1^a^ (0 – 3) |
| *A – wags the tail* | 0^b^ (0 – 1) | 1ª (0 – 1) | 0^b^ (0 – 1) | 1ª (0 – 1) |
| *B – bites the bars or objects* | 0 (0 – 0) | 0 (0 – 1) | 0 (0 – 1) | 0 (0 – 1) |
| *C –head is below the spinal column.* | 0^b^ (0 – 0) | 1^a^ (0 – 1) | 0^b^ (0 – 1) | 0^ab^ (0 – 1) |
| *D –difficulty in overcoming obstacles* | 0 (0 – 0) | 0 (0 – 1) | 0 (0 – 1) | 0 (0 – 1) |

UPAPS: UNESP-Botucatu pig composite acute pain scale. Differences in gold standard pain scores between moments (a > b > c) according to the Friedman test (p < 0.05). M1: preoperative; M2: postoperative, before rescue analgesia; M3: postoperative, after rescue analgesia; M4: 24 hours postoperative; MA - data from all moments grouped (M1 + M2 + M3 + M4).
