# Supplementary figures and images for: Validation of the UNESP-Botucatu pig composite acute pain scale (UPAPS)
Source: PLoS One. 2020 Jun 1;15(6):e0233552. doi: 10.1371/journal.pone.0233552 (PMC7263847; doi:10.1371/journal.pone.0233552)

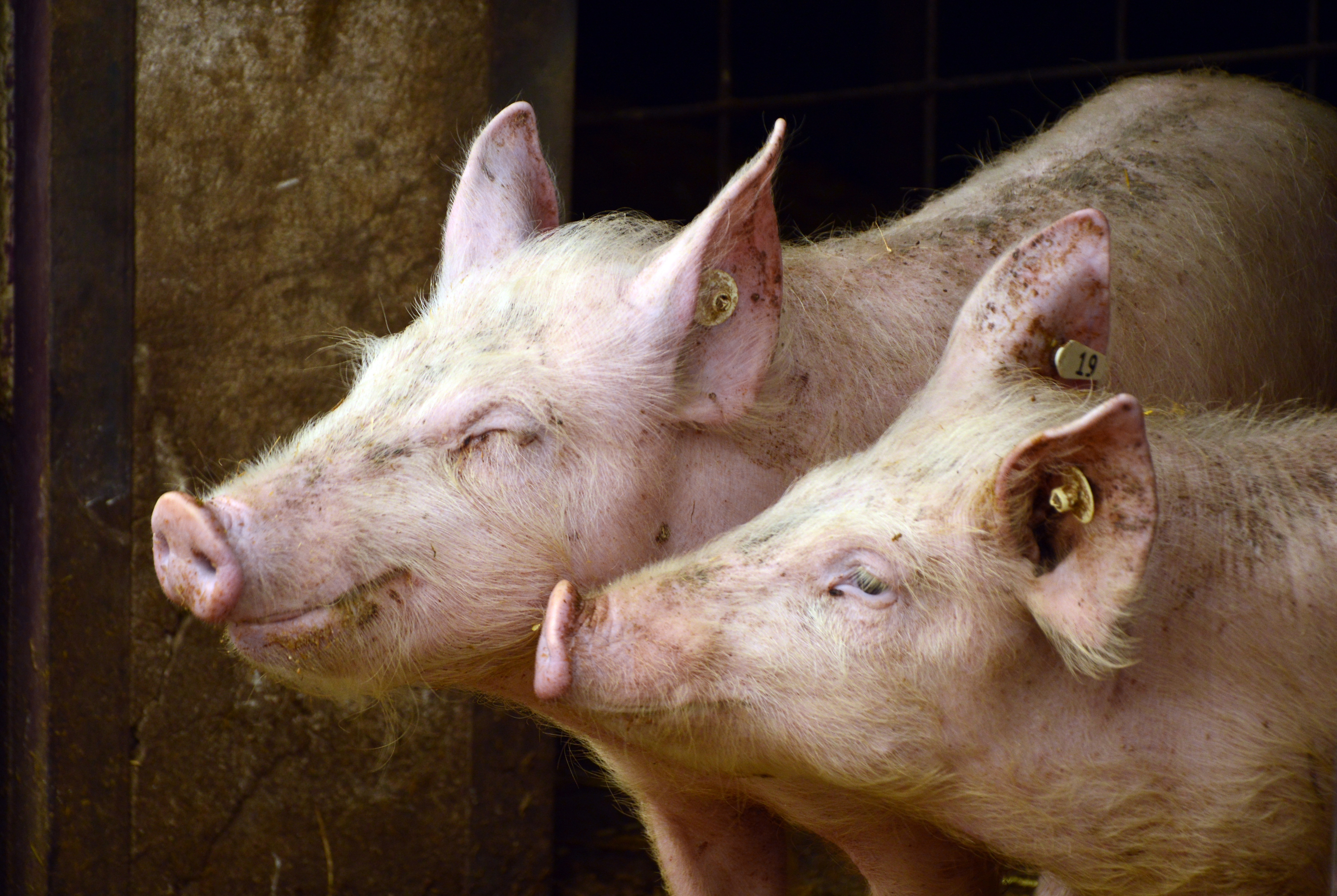

Supplement: S1 Fig — (JPG) [file pone.0233552.s003.jpg]
