# Supplementary material for: Validation of the UNESP-Botucatu pig composite acute pain scale (UPAPS)
Source: PLoS One. 2020 Jun 1;15(6):e0233552. doi: 10.1371/journal.pone.0233552 (PMC7263847; doi:10.1371/journal.pone.0233552)
Supplement: Attachment — Submitted filename: Response to Reviewers.docx [file pone.0233552.s004.docx]

Additional Editor Comments (if provided):

Dear Authors,
it seems that both the reviewer have some minor related to your study. Please provide a complete response or amend the manuscript accordingly their suggestions if possible

*The authors appreciate the reviewers for taking the time to review this manuscript carefully.*

*In the meantime the manuscript was reviewed, the authors developed a further analysis of data. We would appreciate the permission of the editor to include data regarding the predictive criterion validity (Table 8), the Youden indexes of the unidimensional scales (Table 16), the percentage of pigs present in the diagnostic uncertainty zone (Table 17) and finally, the most essential data in our opinion, the classification of pain intensity according non-hierarchical cluster analysis (Fig 6 and 7). The authors believe these inclusions will strengthen the validation process.*

*To compensate these inclusions, the Introduction section and Table 15 were reduced and individual data from Fig 5 were grouped in one graph instead of 4.*

*All corrections were performed as suggested. Please see the comments below.*

Journal Requirements:

When submitting your revision, we need you to address these additional requirements:

1. Please ensure that your manuscript meets PLOS ONE's style requirements, including those for file naming. The PLOS ONE style templates can be found at <http://www.plosone.org/attachments/PLOSOne_formatting_sample_main_body.pdf> and <http://www.plosone.org/attachments/PLOSOne_formatting_sample_title_authors_affiliations.pdf>

*Answer: these links do not seem to work, but we followed instructions for authors*

2. In your Methods section, please provide additional details regarding the animals used in your study and ensure you have described the source. For more information regarding PLOS' policy on materials sharing and reporting, see <https://journals.plos.org/plosone/s/materials-and-software-sharing#loc-sharing-materials>.

*Answer: This information was included (page 6, line 7)*

3. In your Methods section, please include a comment about the state of the animals following this research. Were they euthanized or housed for use in further research? If any animals were sacrificed by the authors, please include the method of euthanasia and describe any efforts that were undertaken to reduce animal suffering.

*Answer: This information was included (page 7 – line 14-17). After the end of the field study pigs were kept until 145 days of age when they were submitted to humane slaughter accomplished by electrical stunning, followed by exsanguination, according to the Brazilian Federal legislation (Ministry of Agriculture).*

4. Please amend the manuscript submission data (via Edit Submission) to include author Flávia Augusta de Oliveira.

*Answer: Flavia Oliveira was included*

5. Please ensure that you refer to Figure 2 and 5 in your text as, if accepted, production will need this reference to link the reader to the figure.

*Answer: corrected*

6. Please upload a new copy of Figure 5 as the detail is not clear. Please follow the link for more information: [http://blogs.PLOS.org/everyone/2011/05/10/how-to-check-your-manuscript-image-quality-in-editorial-manager/](http://blogs.plos.org/everyone/2011/05/10/how-to-check-your-manuscript-image-quality-in-editorial-manager/)

*Answer: All figures were uploaded after using PACE*

7. We note you have included a table to which you do not refer in the text of your manuscript. Please ensure that you refer to Table 2 in your text; if accepted, production will need this reference to link the reader to the Table.

*Answer: included*

8. Please include captions for your Supporting Information files at the end of your manuscript, and update any in-text citations to match accordingly. Please see our Supporting Information guidelines for more information: <http://journals.plos.org/plosone/s/supporting-information>.

*Answer: included*

[Note: HTML markup is below. Please do not edit.]

Reviewers' comments:

Reviewer's Responses to Questions

**Comments to the Author**

1. Is the manuscript technically sound, and do the data support the conclusions?

The manuscript must describe a technically sound piece of scientific research with data that supports the conclusions. Experiments must have been conducted rigorously, with appropriate controls, replication, and sample sizes. The conclusions must be drawn appropriately based on the data presented.

Reviewer #1: Yes

Reviewer #2: Yes

 2. Has the statistical analysis been performed appropriately and rigorously?

Reviewer #1: Yes

Reviewer #2: Yes

3. Have the authors made all data underlying the findings in their manuscript fully available?

The [PLOS Data policy](http://www.plosone.org/static/policies.action#sharing) requires authors to make all data underlying the findings described in their manuscript fully available without restriction, with rare exception (please refer to the Data Availability Statement in the manuscript PDF file). The data should be provided as part of the manuscript or its supporting information, or deposited to a public repository. For example, in addition to summary statistics, the data points behind means, medians and variance measures should be available. If there are restrictions on publicly sharing data—e.g. participant privacy or use of data from a third party—those must be specified.

Reviewer #1: Yes

Reviewer #2: Yes

 4. Is the manuscript presented in an intelligible fashion and written in standard English?

PLOS ONE does not copyedit accepted manuscripts, so the language in submitted articles must be clear, correct, and unambiguous. Any typographical or grammatical errors should be corrected at revision, so please note any specific errors here.

Reviewer #1: Yes

Reviewer #2: Yes

 5. Review Comments to the Author

Please use the space provided to explain your answers to the questions above. You may also include additional comments for the author, including concerns about dual publication, research ethics, or publication ethics. (Please upload your review as an attachment if it exceeds 20,000 characters)

Reviewer #1: This study reports the development and validation of the UNESP-Botucatu composite pain scale for assessing postoperative pain in pigs. It includes an ethogram for the development of the scale and the validation based on video footage from 45 piglets undergoing castration. Four moments were assessed by 4 different observers. Validation of the scale and definition of the cut-off for administration of rescue analgesia were performed based on solid statistics. The study is well designed, conducted and reported.
Most of this reviewer’s comments were made for improving the readability and fluidity of the text as well as for providing more details and clarifying some points of the methodology and results sections.

*The authors appreciate the reviewer for taking the time to review this manuscript carefully.*

*In the meantime the manuscript was reviewed, the authors developed a further analysis of data. We would appreciate the permission of the reviewer to include data regarding the predictive criterion validity (Table 8), the Youden indexes of the unidimensional scales (Table 16), the percentage of pigs present in the diagnostic uncertainty zone (Table 17) and finally, the most essential data in our opinion, the classification of pain intensity according non-hierarchical cluster analysis (Fig 6 and 7). The authors believe these inclusions will strengthen the validation process.*

*To compensate these inclusions, the Introduction section and Table 15 were reduced and individual data from Fig 5 were grouped in one graph instead of 4.*

*All corrections were performed as suggested. Please see the comments below.*

Abstract:
Line 1: Perhaps change “mandatory” to “essential”?
Line 3: change to “treatments”
Line 5: change “submitted to” to “undergoing”
Lines 6-9: Suggest changing to: “Behavior was video-recorded 30 minutes before and intermittently up to 24 hours after castration. Edited footage (before surgery, after surgery before and after rescue analgesia, and 24 hours postoperatively) was analysed twice (one month apart) by one observer who was present during video-recording and three blinded observers”.
Line 13: Suggest changing to: “except between observers 1 and 3 (moderate agreement; 0.57)”
Line 14: Remove “the” from “based on the principal component”

Answer: *All corrections were performed.*

Introduction:
The Introduction is very nice and it touches the most important points pertaining to the study. However, it is overly long. Please edit the introduction to shorten the amount of text.

*Answer: Introduction section was reduced of about 25%.*

Page 3, Line 7: Change to: “…respectively. However, among..”
Page 3, Lines 15-16: Please clarify that the data on beef and dairy cattle refer to animals undergoing the same procedure (castration?).
Pages 3 and 4, Lines 24-2: Suggest changing to: “This paradigm has been challenged by the fact that pigs treated with anaesthesia and analgesia showed increased short- and long-term weight gain when compared with pigs castrated without anaesthesia; thus, providing an economic benefit to producers.”
Page 4, Lines 6-8: Please split this sentence in two (one for Canada and another for USA and Europe).
Page 4, Line 21: Change “determination” for “assessment”
Page 5, Lines 4-13: Suggest deleting this paragraph. It does not add much relevant information.
Page 6, Line 4: Please correct: “this species” (singular)
Page 6, Lines 1-14: There is a link missing between these paragraphs. Please edit this section to shorten and link them.
Page 5, Lines 20-24 and Page 6, Lines 15-18: These two sections should be together.

Answer: *All corrections were performed and introduction was condensed.*

Materials and methods
Page 7, Line 12: Was food also available ad libitum? Please clarify

*Answer: Information was included. They were fed three times a day and food was also offered during the perioperative pain assessment moments (page 6, line 15; oage 7, line 12-14)).*

Page 7, Line 14: Please remove the comma “..maternity period when piglets”
Page 7, Lines 14-16: Was the observer actively making physical contact with the piglets in the first week? Please clarify what you mean by “friendly contact with the piglets”. Also, I am not sure what you mean by “the observer followed the daily routine of the sector”.

*Answer: more detail was included to make things clearer. “After a week of contact with the piglets, by cleaning the pen and providing food, the in-person researcher initiated direct friendly contact inside the stall, for 15 minutes three times a day, without making any sudden movement, or using vocal communication, so that the piglets could spontaneously approach” (page 6, line 18-21. The pigs by this stage were playing with the in-person researcher, by smelling, nibbling and pulling clothes and hair*.

Page 7, Line 22: 1 mL divided into two doses of 0.5 mL into each testicle? Please clarify.

*Answer: This was clarified. Pigs received 0.5 mL of 1% lidocaine at each incision line, and 1 mL injected intratesticularly at each testicle (page 6, line 24-25 and page 7, line 1).*

Page 8, Line 8: When you say “evaluator”, I assume you refer to the observer who had been familiarizing with the pigs. It does not seem that this individual was actually ‘evaluating’ pain, correct?

*Answer: The local (in-person) observer was replaced by in-person researcher to avoid misinterpretation throughout the manuscript.*

Please clarify the presence of the observer. Was it only to set the camera? Was he/she the one evaluating the surgical wound and/or giving the medications?

*Answer: The local (in-person) observer was replaced by in-person researcher to avoid misinterpretation throughout the manuscript.*

Please also specify the sex of this individual and the other 3 observers.

Answer: included. One man and three women.

Page 8, Lines 10-17: How long were the selected footage for each moment? I assume the individual who edited the footage was the same familiarizing with the pigs and doing the video recording?

*Answer: This was included in line 10-11 (Page 7). This information was included again in line 17, page 8.*

Also, you might want to use “;” instead of “,” between moments and put explanations between parenthesis to facilitate the reading since this sentence has 7 lines. Consider removing the information being repeated in Figure 1.

*Answer: This sentence was removed and all information included in Figure 1 as suggested.*

Page 9 Line 5: Do you mean “behaviours observed in the pilot study?”. It seems like there is something missing here.

*Answer: “The scale was developed according to the analysis of the relevance of pain-related behaviours previously described in the literature[6,8,32-35] and behaviours observed both in the pilot study and during video edition by the in-person researcher.” (page 8, line 4-6)*

Page 9 Line 5: “were accepted”. Do you mean that they were included in the scale?

*Answer: Yes. Corrected*

Page 9 Lines 13-16: What do you mean by “variable counting scale”?

*Answer: excluded because this is irrelevant and to avoid misinterpretation.*

Page 9 Lines 17-20: Please remove. You already partially said this. No need to repeat. Only clarify on page 8 lines 10-17 that the edited videos were each 4 minutes long.

*Answer: partially deleted (page 8, line 17-18)*

Page 11 Lines 12-13: Description of the Altman’s classification should be reported in line 3 since this is the first time you refer to it.

*Answer: corrected*

Results
Table 1: I don’t see the explanation for the Asterix. Please include it.

*Answer: asterisks were deleted as they mean nothing*

The behavior “rubbing the affected area on the floor” is described under ‘Altered posture’ and ‘Lie down uncomfortably’. How did the observer choose to classify this behavior since it is described under two different categories?

*Answer: Please accept our apologies for this mistake. This behaviour was classified as ‘altered posture’ when pigs rubbed the affected area against any structure (like the grid) only when standing; therefore ‘on the floor’ was deleted from here. Otherwise, when pigs rubbed the affected area specifically on the floor when they were lying down, this behaviour was included in the item ‘Lie down uncomfortably’. For the item ‘Attention to the affected area’ of the scale, the behaviour ‘scratches/rubs the painful area’ was scored both when pigs scratched on the floor or against any surface, like the grid.*

Table 2: “percentage presence”? Please reword.

*Answer: corrected*

Please explain the superscript letters.

*Answer: included*

Page 18 Line 5: Correct “Table 2”. Not “Table 1”.
Table 3: Excellent idea to have the videos in the table! Please recheck the links as some videos are not working.

*Answer: The authors acknowledge your comment. We rechecked again and they are all working in Google Chrome and Internet explorer. If the reviewer copy and paste straight to the browser they should work. Could the reviewer please be kind enough to let us know which link is not working?*

Table 4: You already explained in the title that values are reported as ICC (CI), so perhaps you can consider deleting the second line of the table [ICC (CI].

*Answer: deleted*

If the ICC used for evaluating criterion validity is the ICC between the gold standard and the other observers, how come the ICC between gold standard and gold standard is 0.88? I would expect it to be 1. The same would be for ICC between Observer 1 and Observer 1, and so on. It might be that I don’t understand the statistics of this, but regardless, it’s probably a good idea to explain it a bit more.

*Answer: Please accept our apologies. We double checked the data. The inter-observer data were correct, but the intra-observer was not. This was amended and duplicate data were removed to facilitate reading (now this is Table 6 as Criterion validity data were placed together, according to Reviewer 2 suggestion)*

Criterion validity would only consider the ICC between the gold standard and each of the other observers, correct? In that case, do you need to report the ICC between observers 1, 2 and 3?

*Answer: If the reviewer agrees, the authors think it is interesting to inform the correlation among all observers for comparison as well, however as mentioned before duplicate data were removed to facilitate reading (now this is Table 6 as Criterion validity data were placed together, according to Reviewer 2 suggestion).*

Table 8: Please add the explanation of the Asterix in the table legend.
Perhaps it would be nice to also explain what these ‘dimensions’ are in the legend.

*Answer: Now table 9. Asterisk was included in the legend, and so was additional information regarding this analysis. Please see the explanation of the legend of Figure 2 as well.*

Page 26, Line 1: Please remove ‘the’ from “analysis at the four”.

*Answer: corrected*

Table 11: In Page 10, Lines 6-7, it is not clear that the scores used for definition of the cut-off for rescue analgesia were only the scores from the second phase of video analysis. Please clarify.

*Answer: The authors presume the reviewer is referring to Table 14 (now Table 15)? This information was included. Only one ROC curve by grouping all observers replaced the individual ROC curves.*

Page 32 Lines 9-11. This sentence is confusing. Please reword.

*Answer: Amended (page 30, line 4-11).*

Discussion
Page 37 Line 4: Correct “this species”
Page 37 Line 22: Remove “only”
Page 38 Line 5: Consider changing “tool is carried out” for “tool should be carried out”

*Answer: Amended*

Page 38 Lines 17-20: This sentence is confusing. Please rephrase.

*Answer: The sentence was excluded, and the paragraph was shortened. Content validation was not only based on “expert” opinion, but also included the ethogram findings and behaviour described in the literature (page 36, line 10-14).*

Page 38 Lines 22-24: Do you mean that the observers were selected because they had experience in pain scoring in cattle? This section is not clear. Indeed, detailed information on the sex and level of training of observers is not clear in the M&M. I even wonder if you need to discuss them here or if clearly explaining them in the M&M is enough. I’ll leave this to your discretion.

*Answer: More detailed information regarding the observers were include in Methods section (page 9, line 1-6) and the paragraph was rephrased (page 36, line 2-5).*

Page 39 Lines 4-8: You used this as an additional means of assessing construct validity, correct? In that case, this paragraph should be linked to Page 38 Lines 3-11. It seems a bit lost herein.

*Answer: Thanks for pointing that. This part is related to criterion validity. These sentences were placed together and followed the order of results to maintain consistency (page 36, line 15-24, page 37, line 1-2.*

Page 39 Lines 16-17: I don’t see a link for the sentence “Therefore, … factor analysis”. Please reword or delete this sentence.

*Answer: deleted*

Page 41 Lines 6-9: In this sentence you refer to production and research animals. But the sentence is constructed in a manner that only refers to production animals. Please reword.

*Answer: the paragraph was shortened and rephrased (page 39, line 1-7).*

Page 42 Line 2: Suggest changing to: “pain-related behavior changes were more evident”

*Answer: corrected (page 40, line 7)*

Page 42 Lines 6-12: Perhaps add a small sentence related to the advantage of video analysis as the presence of the observer might affect pain-related behaviors?

*Answer: included (page 40, line 10-18)*

Reviewer #2: Dear Authors,
thank you very much for your paper. it covers a very useful topic and is very well conducted, despite the huge amount of data analyzed and discussed.
I have only a few suggestions, that I attach below:

*The authors appreciate the reviewer for taking the time to review this manuscript carefully.*

*In the meantime the manuscript was reviewed, the authors developed a further analysis of data. We would appreciate the permission of the reviewer to include data regarding the predictive criterion validity (Table 8), the Youden indexes of the unidimensional scales (Table 16), the percentage of pigs present in the diagnostic uncertainty zone (Table 17) and finally, the most essential data in our opinion, the classification of pain intensity according non-hierarchical cluster analysis (Fig 6 and 7). The authors believe these inclusions will strengthen the validation process.*

*To compensate these inclusions, the Introduction section and Table 15 were reduced and individual data from Fig 5 were grouped in one graph instead of 4.*

*All corrections were performed as suggested. Please see the comments below.*

INTRODUCTION

Lines 15-16 (pag. 5): I would eliminate the sentence: Providing that a pain-related behaviour scale is validated, the instrument should present reliability, specificity, and sensitivity.

*Answer: deleted as requested*

Line 24 (pag. 5): for cats, I would add the reference by Reid et al.: Definitive Glasgow acute pain scale for cats - validation and intervention level

*Answer: included*

METHODS

Lines 24 (pag 6)-2 (pag 7): I would move the sentence: “A pilot study was carried out with 12 piglets aged 35 days, submitted to castration to define the experimental protocol. For the main study, 45 Landrace, Large White, Duroc, and Hampshire male pigs were randomly selected. The pigs were aged 1 38±3 days (range2 35 – 41 days) and weighed 11.06 ± 2.28 kg” after the inclusion and exclusion criteria at the end of line 8 (pag. 7)

*Answer: changed as requested (page 6, line 7-11).*

Line 17 (pag. 8): move here, after “(M4)”, the citation to fig. 1

*Answer: The description of moments were maintained only in Fig 1 as requested by Reviewer 1. The citation of Fig 1 was inserted in line 20, page 7.*

Line 19: eliminate citation to fig. 1 here

*Answer: excluded*

Line 8 (page 10): the sentence should be changed as follow: Subsequently, they were required to ascribe a score for the developed scale as well as the visual analogue scale (VAS), numerical rating scale (NRS), where….

*Answer: We apologize but it is necessary to maintain as before as the observers first scored the unidimensional scales and then the proposed scale. We condensed the explanation about the scores and placed between brackets (page 9, line 8-14).*

STATISTICAL ANALYSIS

Line 21-22 (pag. 10): As here you are talking about inter-observer reliability and not about criterion validity, I would change the sentence: “comparing the gold-standard assessments against the other observers respectively” in “comparing the degree of agreement among different observers”.

*Answer: amended (page 9, line 22-23)*

Lines 12-13 (pag. 11): I would delete here the specifications about the Altman’s classification and move it at the end of line 3.

*Answer: corrected*

Lines 18-20 (page 11): Is this sentence correct?

*Answer: Corrected to “The factorial structure was confirmed when items showed a factor load ≥ 0.50 or ≤ -0.50” (page 10, line 24-25).*

Line 2 (pag 13): delete “S” before “< 70%”

*Answer: corrected*

RESULTS:

Tab 1: What “*” means?

*Answer: asterisks were deleted as they mean nothing*

Tab. 2: the legend should read: “Median and amplitude of the presence (number of minutes of each behaviour/30 minutes) of normal behaviours,….

*Answer: corrected*

Line 5 (pag. 18): eliminate the citation to Table 1 here. At the end of the paragraph (line 10) you should cite Table 2.

*Answer: corrected*

Line 1-11 (pag 21): the title and the paragraph, as well as table 4, is a bit confusing: in the title you put altogether intra- and inter-observer reliability and criterion validity, in the text you mix criterion validity (first part of the sentence (lines 3-4) with inter-observer reliability (lines 4-5) and you do not mention at all intra-observer reliability.
Later on, you have dedicated paragraphs on Intra-observer reliability and Criterion validity (see my comments to line 9 of pag 22).

*Answer: Thanks for pointing that. Table 4 was changed to Table 6 and positioned in Criterion validity section to keep consistency*.

Line 9 (pag. 22) the title of the paragraph should read “Criterion validity”!

*Answer: corrected as described above. Matrix correlation is Criterion validity*

Line 1 (pag. 23): the Title of table 6 should refer to criterion validity and not to inter-observer reliability

*Answer: Table 6 (now Table 5) is not Criterion validity, therefore table 4 (now Tale 6 - Matrix coefficient) was positioned in Criterion validity section to maintain coherence, as previously described.*

Line 10 (pag. 23): I Would keep in the title only “Concurrent validity”

*Answer: changed to Criterion validity because a new data were incorporated, therefore, criterion validity was subdivided in concurrent and predictive criterion validity page 21, line 6).*

Line 12 (pag. 25): fig. 2 is not cited in the text. Maybe you can cite it together with Table 8 (pag. 24)

*Answer: included*

Line 12 (pag. 27): move “(table 10)” at the end of the following sentence (line 14).

*Answer: replaced*

Line 19 (pag. 32): fig. 4 is not cited in the text. Maybe you can cite it together with Fig. 3 (line 11)

*Answer: Fig. 3 and 4 were cited in the first sentence (page 30, line 4)*

Line 4 (pag. 33): it should read “Fig 5”!?!

*Answer: corrected*

DISCUSSION

Line 8 (pag. 38): I would refer to “construct validity” and not to concurrent validity as you are referring to the comparison with the “gold standard” and not with VAS, NAS and SDS.

*Answer: In this study there were two approaches to perform concurrent criterion validity. One was by comparing the scale against the unidimensional scales and the other one was to compare the gold standard assessment against the other observers. Construct validity is related to responsiveness (Streiner DL, Norman GR, Cairney J. Health measurement scales: a practical guide to their development and use. 5th ed. Oxford: Oxford University Press; 2015)*

Line 5 (pag. 39): in order to avoid confusion, I would use the term “concurrent validity” instead of “reliability”.

*Answer: Thanks for your suggestion. This was corrected (page 36, line 23).*
