# Supplementary material for: Validation of the UNESP-Botucatu pig composite acute pain scale (UPAPS)
Source: PLoS One. 2020 Jun 1;15(6):e0233552. doi: 10.1371/journal.pone.0233552 (PMC7263847; doi:10.1371/journal.pone.0233552)
Supplement: Attachment — Submitted filename: Response to Reviewers.docx [file pone.0233552.s005.docx]

Answers to reviewers

Reviewers' comments:

Reviewer #1: Thank you for addressing the concerns raised in the first review. The manuscript has much improved and there are only minor comments now. The main thing I think is still missing is a clear description of what constituted the pilot study and what constituted the validation study. The authors are congratulated on advancing pig welfare with this research.

Answer: the authors are very grateful for the suggestions which were fully incorporated.

Page 2 Line 12. Please removed ‘of’ from: “most of observers”

Answer: corrected (page 2, line 13)

Page 2 Lines 18-19: Please reword: “increase and decrease in pain scores of all items of the scale after castration and intervention analgesia, respectively, according to..)

Answer: corrected and rephrased (page 2, line 18-19)

Page 6 Line 24: I would assume you need to specific the manufacturer for the lidocaine. Please check the Journal’s instructions. Same for the other drugs.

Answer: included (page 7, line 6-7, 12, 15-16)

Page 7 Lines 18-19 Is this the pilot study? Please clarify

Answer: “from the main study” was included (page 8, line 3)

Page 7 Lines 21-22: Replace “time in seconds spent on” by “duration”

Answer: corrected (page 8, line 7)

Fig 1 legend: Reword: “..moments used for validation”

Answer: corrected to “Timeline of interventions, drugs and pain assessments performed for validation of the Unesp-Botucatu pig composite acute pain scale (UPAPS)” (page 8; line 9)

Page 8 Line 17 Please reword “The in-person researcher edited each of the four 30-minute videos into 4-minute videos representing the 4 moments..”

Answer: corrected (page 9, line 1-2)

Page 9 Line 3: Change to ‘Developed”

Answer: corrected (page 9, line 5)

Page 10 Line 12: “confidence interval” – You already defined this abbreviation on line 1 of the same page.

Answer: ‘confidence interval’ was excluded from here

Page 13 Lines 10-13: This is still a bit confusing, and I think it is because it is not entirely clear from M&M what was the pilot study for development of the ethogram and what was the validation study. Please make sure this is clear in the M&M and in the results. Consider rewording this sentence.

Answer: “A pilot study was carried out with 12 piglets aged 35 days, submitted to castration only to define the experimental protocol and to recognize pain-related behaviours.” (page 6; line 15-16);

“Footage from 40 out of 45 pigs was used for the ethogram because in 5 pigs footages were recorded for slightly less than 30 minutes at some moments; therefore data from these 5 five pigs were excluded from the ethogram analysis. For validation of the scale, 180 videos of approximately 4-minute durations were edited and footage of the main study from 45 pigs were used” (page 13; line 17-21).

Page 16 Line 7-9: The words “after surgery” should be at the end of the sentence in line 9.

Answer: corrected (page 16, line 9)

Page 16 Line 13-14: In Page 8 line 13, you refer to ‘six behavioral categories’, here you refer to ‘items’. Please standardize the wording.

Answer: ‘categories’ was replaced by ‘items’ in all cases

Table 3. Posture (3); Interaction (0, 1,2,3); Activity (0, 2, 3); Appetite (0,1,2,3); Attention (B, C,D); Miscellaneous (A, B, C, D): the link does not work, but copying and pasting the web address works.

Answer: all links are working correctly now without the need to copy and paste. We had to press ‘enter’ after the link to work out.

Table 13 – Is this table really necessary in the manuscript? Perhaps as supplementary material?

Answer: Table 13 was excluded from the main text and included as supplementary material (S1 Table) as required (page 28, line 9)

Page 36 Line 4: ‘before the observations’

Answer: corrected (page 37, line 5)

Page 37 Line 1: You are repeating the same information from line 18 in the previous page

Answer: “and this methodology has already been used in other studies in cats [21], cattle [22], and horses[27]” was excluded

Reviewer #2: Dear Authors, thanks so much for your revision. Now the paper sounds really good!!
I only have remained 2 small observations, but it's up to you if make the changes or not (in the case I have misunderstood).

The authors are very grateful for the previous and present suggestions which were fully incorporated.

1) In M&M you report that video-observers are asked to state if they would provide rescue analgesia and to ascribe a score for VAS, NAS and SDS, but you don't report that they also had to score the UPAPS!!.
So, I would read line 9-12 (page 9) as: "they were required to ascribe a score for the visual analogue scale (VAS), numerical rating scale (NRS; 0 “no pain” to 10 “worst possible pain”), and the simple descriptive scale (SDS; 0 - no pain to 3 - intense pain), AND THEN TO SCORE THE PROPOSED SCALE.

Answer: thanks for addressing that. This was included (page 9, line 14).

2) In my experience, Table 5 shows results for Concurrent criterion validity (i.e. comparison between the gold standard and each observer), and not for inter-observer reliability that, as stated in lines 22-23 (page 9) refers to the degree of agreement among observers. It's true that also the agreement between the gold standard and each single observer can be part of the inter-observer reliability, but in this table data showing inter-observer reliability (comparison among the 3 observers) are non shown... Indeed, in the first line of table 6 (that is cited in the text as showing data for criterion validity), you repeat results shown in table 5... And I'm still not able to understand the following 2 lines of table 6... are you comparing here data among observers? And if yes, why Observer 2 is not matched with Observer 1?
Still a bit confusing...

Answer: Although both Tables 5 and 6 report results of inter-observer reliability, the authors agree that for the concurrent criterion validity, results from the Gold standard should be compared to the other observers; therefore Tables 5 and 6 were swapped to consider this suggestion. We hope it is clearer now.

Table 5 (previous Table 6) expresses agreements among all observers. The comparison between Observer 1 and 2 is in the intersection between the 3^rd^ line and 3^rd^ column of table 5 (previous table 6).
